# Cationic Liposomes: A Flexible Vaccine Delivery System for Physicochemically Diverse Antigenic Peptides

**DOI:** 10.1007/s11095-018-2490-6

**Published:** 2018-09-12

**Authors:** Jeroen Heuts, Eleni Maria Varypataki, Koen van der Maaden, Stefan Romeijn, Jan Wouter Drijfhout, Anton Terwisscha van Scheltinga, Ferry Ossendorp, Wim Jiskoot

**Affiliations:** 1 Department of Immunohematology and Blood Transfusion, Leiden University Medical Centre, P.O. Box 9600, 2300 RC, Leiden, The Netherlands; 2 Division of BioTherapeutics, Leiden Academic Centre for Drug Research (LACDR), Leiden University, P.O. Box 9502, 2300 RA Leiden, The Netherlands; 3 Department of Clinical Pharmacy and Toxicology, Leiden University Medical Centre, Leiden, P.O. Box 9600, 2300 RC, Leiden, The Netherlands

**Keywords:** cationic liposomes, immunogenicity, neoepitope, synthetic long peptides, therapeutic cancer vaccine

## Abstract

**Purpose:**

Personalized peptide-based cancer vaccines will be composed of multiple patient specific synthetic long peptides (SLPs) which may have various physicochemical properties. To formulate such SLPs, a flexible vaccine delivery system is required. We studied whether cationic liposomes are suitable for this purpose.

**Methods:**

Fifteen SIINFEKL T cell epitope-containing SLPs, widely differing in hydrophobicity and isoelectric point, were separately loaded in cationic liposomes via the dehydration-rehydration method. Particle size and polydispersity index (PDI) were measured via dynamic light scattering (DLS), and zeta potential with laser Doppler electrophoresis. Peptide loading was fluorescently determined and the immunogenicity of the formulated peptides was assessed in co-cultures of dendritic cells (DCs) and CD8^+^ T-cells *in vitro*.

**Results:**

All SLPs were loaded in cationic liposomes by using three different loading method variants, depending on the SLP characteristics. The fifteen liposomal formulations had a comparable size (< 200 nm), PDI (< 0.3) and zeta potential (22–30 mV). Cationic liposomes efficiently delivered the SLPs to DCs that subsequently activated SIINFEKL-specific CD8^+^ T-cells, indicating improved immunological activity of the SLPs.

**Conclusion:**

Cationic liposomes can accommodate a wide range of different SLPs and are therefore a potential delivery platform for personalized cancer vaccines.

**Electronic supplementary material:**

The online version of this article (10.1007/s11095-018-2490-6) contains supplementary material, which is available to authorized users.

## Introduction

Therapeutic cancer vaccines aim to amplify a specific cellular immune response directed towards the patients’ own tumor (1,2)**.** T-cells are able to identify and destroy malignant cells through the recognition of tumor specific antigens. Vaccination with synthetic long peptides (SLPs) containing a cytotoxic (CD8^+^) as well as a helper (CD4^+^) T cell epitope has shown to induce tumor specific T cell responses that were able to control or even regress tumor outgrowth (1–7). In order to provoke such functional immune responses, the SLPs have to be delivered to and taken up by dendritic cells (DCs), and be processed and presented on MHC molecules to activate tumor specific T cells. However, SLPs by themselves are poorly immunogenic due to inefficient uptake by DCs, resulting in low levels of antigen presentation and subsequent T cell activation (1,4,7,8). A proper formulation of the SLPs with adjuvants and a delivery vehicle is essential to compose an adequate immunogenic SLP vaccine (1,4,5,7,8).

Recently, cationic liposomes have shown to be a promising vaccine delivery platform for therapeutic cancer vaccines, as they were able to increase the immunogenicity of antigen-based vaccines (4,7–10). We have previously shown that cationic liposomes are suited to encapsulate several different SLP sequences of the ovalbumin model protein and of the oncogenic protein E7 of human papilloma virus. The liposome-encapsulated SLPs efficiently induced functional antigen-specific CD8^+^ and CD4^+^ T cells, and were able to induce T cell mediated tumor regression and immunological protection in two different tumor-bearing mouse models (4,7). Liposomally formulated SLPs were effective at a highly reduced dose as compared to the SLPs emulsified in Montanide (~65 fold lower dose), while still resulting in efficient tumor killing in mice (4).

The latest advancements in cancer research have shown the potential of a new class of highly specific tumor antigens. These antigens arise from somatic DNA mutations and result in the expression of mutated peptides on the cancer cell surface that are not present on healthy cells, the so-called neoepitopes (1,2,5). Multiple mutated peptide sequences can be presented by tumor cells, which are by definition the ideal cancer-specific vaccination targets but have originated from random DNA mutations and are therefore patient specific. Therefore, a therapeutic personalized peptide-based vaccine will contain a unique set of mutated epitope-harboring SLPs with various physicochemical properties (1,2,5). For that reason a generic delivery system for neoepitope based vaccines should be able to accommodate a wide variety of different SLPs. The formulation strategies for such a system need to be adjustable to variable SLP characteristics. At the same time we aimed to limit the number of formulation conditions to circumvent time-consuming formulation development for every newly discovered neoepitope peptide sequence.

In the current study we investigated the feasibility of cationic liposomes as a delivery system for SLP based cancer vaccines. By designing 15 different model SLPs with a wide range of physicochemical properties, in particular with respect to isoelectric point (pI) and hydrophobicity, all harboring the SIINFEKL epitope for immunological validation, we developed three different formulation methods to encapsulate all these different peptides into liposomes. The physicochemical characteristics of the liposomes were determined and in an *in vitro* setting we confirmed the retained antigenic properties of the SIINFEKL T cell epitope.

## Materials and Methods

### Materials

The fluorescently labeled as well as the non-fluorescently labeled 24-mer SLPs (Table I) including the immunodominant cytotoxic T lymphocyte epitope [SIINFEKL] of ovalbumin were synthesized. The lipids DOPC and DOTAP were purchased from Avanti Polar Lipids (Alabaster, Alabama, USA). Acetonitrile (ACN), chloroform (CHCl_3_), and methanol (MeOH) were obtained from Biosolve BV (Valkenswaard, the Netherlands) and Vivaspin 2 centrifuge membrane concentrators were purchased from Sartorius Stedim Biotech GmbH (Gӧttingen, Germany). Iscove’s modified Dulbecco’s medium (IMDM, Lonza Verniers, Belgium) containing 8% (*v*/v) fetal calf serum (Greiner Bioscience, Alphen a/d Rijn, the Netherlands) and 50 μM β-mercaptoethanol (Sigma-Aldrich, Zwijndrecht, the Netherlands) was supplemented with either 2 mM Glutamax (Thermo Fisher, Bleiswijk, the Netherlands) and 80 IU/ml sodium-penicillin G (Astellas, the Netherlands) for D1 cells or with 100 IU/ml penicillin/streptomycin, 2 mM glutamin (Thermo Fisher, Bleiswijk, the Netherlands) and 500 μg/ml Hygromycin B (AG Scientific, San Diego, USA) for the B3Z cells. NP-40, chlorophenol red-β-galactopyranoside (CPRG) and dimethyl sulfoxide (DMSO) were obtained from Sigma-Aldrich (Zwijndrecht, the Netherlands). Deionized water with a resistivity of 18 MΩ•cm was produced by a Millipore water purification system (MQ water). Phosphate buffer (PB) was composed of 7.7 mM Na_2_HPO_4_^.^ 2 H_2_O and 2.3 mM NaH_2_PO_4_^.^ 2 H_2_O, (10 mM PB, pH 7.4). MQ water and 10 mM PB, pH 7.4, were filtered through a 0.22 μm Millex GP PES-filter (Millipore, Ireland) before use. Phosphate-buffered saline, (PBS: 140 mM NaCl, 8.7 mM Na_2_HPO_4_^.^ 12 H_2_O, 1.8 mM NaH_2_PO_4_^.^2 H_2_O, pH 7.4), which was used for the *in vitro* MHC class I assays, was purchased from B.Braun (Meslungen, Germany).

**Table I:** Peptides used in this Study and their Calculated Theoretical Isoelectric Point and Hydropathicity

Peptide ID	Peptide sequence NBD-G-XXXXXXXXXXX-SIINFEKLAAK	Theoretical pI*	Hydropathicity (GRAVY)^**^
SLP 1	DEDKDKDDEEA	3.95	−1.208
SLP 2	DEEEKEGKEKA	4.51	−1.096
SLP 3	RKDDKDDKDLA	6.67	−0.962
SLP 4	RKHDHEHEHHA	7.93	−1.171
SLP 5	EDKKKSEKESA	9.28	−1.017
SLP 6	DEKRKKERELA	9.42	−1.004
SLP 7	DELYDLYDELA	4.13	−0.079
SLP 8	DEGLLRHLDEA	4.99	−0.163
SLP 9	DAKHDHLLHAA	7.53	−0.104
SLP 10	LDKKLLEKELA	8.61	−0.008
SLP 11	RIDIRLIIEIA	8.83	0.713
SLP 12	GSAAESASGSA	6.53	0.126
SLP 13	RDKSLKELLSA	9.54	−0.113
SLP 14	ELIDIIDIEIA	4.25	0.796
SLP 15	DLKLADLLALA	6.44	0.771

*The pI was calculated for the unlabeled peptide of which the N-terminus was not blocked, so the real theoretical pI values are expected to be slightly lower for the labeled peptide

**The sum of hydropathy values of all amino acids, divided by the number of residues in the sequence (the NBD label was not taken into account.). SLPs with a GRAVY score < 0 are relatively hydrophilic and SLPs with a GRAVY score > 0 are relatively hydrophobic (12,15)

### Preparation SLP Loaded Liposomes

Cationic liposomes (DOTAP:DOPC, 1:1 M ratio) containing the SLPs were produced on a small scale, 500 μl – 2 ml per batch, by making use of the thin film dehydration-rehydration method as previously described (4,7). For the separate encapsulation of the SLPs a total of three different encapsulation solvents were utilized which were dependent on the physicochemical properties of the SLPs (Table I). SLPs 1–6 were dissolved in ACN/H_2_O (1:1, v/v), SLPs 7–10 in CHCl_3_:MeOH:H_2_O (60:36:4, v/v) and SLPs 11–15 in 0.04% NH_4_OH (*w*/*v*). SLPs 11 and 13 were diluted from a concentrated DMSO stock, while all other SLPs were dissolved directly from their lyophilized form. SLPs 1–6 and 11–15 were added during the rehydration of the dry lipid film while the SLPs 7–10 were mixed with the lipid stock solutions. After SLP loading and hydration of the dry lipid film, the suspension was freeze dried overnight in a Christ alpha 1–2 freeze dryer (Osterode, Germany). The following day the lipid cake was rehydrated with PB in three consecutive steps: twice the addition of 25% of the final volume (30 min equilibration after each addition) and as a third step the remaining 50% of the final volume was added (followed by 1 h equilibration). Down-sizing of the obtained liposomes was done via extrusion with a Lipex extruder (Northern Lipids Inc., Canada), the particles were extruded four times through a 400 nm and four times through a 200 nm polycarbonate filter (Nucleopore Milipore, Kent, UK). After extrusion the SLP-containing liposomes were separated from the non-encapsulated SLP and concentrated by making use of Vivaspin 2 centrifugation concentrators (molecular-weight-cut-off of 300 kDa), as described previously (4,7). The liposomal dispersions were concentrated 5-fold by centrifugation at 931 g (2000 rpm). Subsequently, the formulation was re-diluted with PB to its initial volume after which the concentration step was repeated. During purification, samples of the liposomal fraction and the flow-through were taken to determine free and encapsulated peptide, as described below.

### Liposome Characterization

#### Physicochemical Properties and Stability of Liposomal Formulations

The hydrodynamic diameter (Z-average) and the polydispersity index (PDI) were determined by using dynamic light scattering (DLS). The zeta-potential was determined by using laser Doppler electrophoresis. Both measurements were performed on a Zetasizer Nano (Malvern Instruments, Malvern, UK) and prior to analysis the samples were diluted 400 fold in PB. The physicochemical properties of all liposomal formulations were determined at the day of production and after 8 weeks to determine liposome stability.

#### Loading Efficiency and Drug Loading of Fluorescent SLPs

The amount of liposomally encapsulated SLP in each formulation was quantified via the fluorescent signal of the NBD group (λ _excitation_ = 462 nm, λ _emission_ = 540 nm). In order to calculate the loading efficiency and total drug loading of the SLPs the liposomal samples and filtrates (containing the non-encapsulated SLP) were collected during the purification/concentration steps. Calibration curves were prepared for all SLPs. In order to exclude possible interference due to the presence of lipids a series of empty liposomes spiked calibration curves were prepared as well. All measurements were performed in 1:1 (v/v) MeOH/PB and data was acquired with a fluorescence micro plate reader (Tecan, Salzburg, Austria). For every formulation the loading efficiency (Eq. 1) and peptide recovery were calculated (Eq. 2).

1 $$ Loading\ efficiency\ \left(\%\right)=\left(\frac{Total\ peptide- free\ peptide\ }{Total\ peptide}\right)x\ 100\% $$

In equitation 1 the *total peptide* accounts for the total amount of SLP in the liposomal dispersion prior to purification. The *free peptide* is the amount non-encapsulated SLP that was determined after purification.

2 $$ Peptide\ recovery\ \left(\%\right)=\left(\frac{Encapsulated\ peptide\ }{Total\ added\ peptide}\right)x\ 100\% $$

In equitation 2 the *encapsulated peptide* accounts for the amount of SLP in the purified and concentrated liposomal dispersion. The *total added peptide* accounts for the amount of SLP that was added during liposome formulation.

#### Liposomal Loading of Non-fluorescent SLPs

The liposomal loading of the three SLPs without fluorescent label was determined by making use of a modified Bligh and Dyer extraction followed by reversed-phase UPLC analysis (see 2.3.4, below) (7). Thirty μl of liposomal formulation was diluted in 100 μl MQ water, followed by the addition of 250 μl methanol and 150 μl chloroform. Subsequently, the mixture was vortexed for ca. 5 s. Next, 250 μl 0.1 M HCl and 125 μl chloroform were added and the resulting mixture was vortexed for ca. 5 s. Finally, the mixture was centrifuged at 233 g (1000 rpm) for 5 min and the upper phase was collected for SLP quantification. The extraction efficiencies of the SLPs were determined by spiking empty liposomes, 10 mg/ml, with a 1 mg/ml solution of the respective SLPs.

#### Peptide Content

The peptide content in the upper phases of the extraction was determined via reversed-phase UPLC (Waters Acquity UPLC^®^ with a waters C18–1.7 μm (2.1 × 50 mm) column). A flow rate of 0.5 ml/min was used with initially 95% solvent A (ACN with 0.1% TFA) and 5% solvent B (MQ with 0.1% TFA), followed by a linear gradient to 79% solvent B in 3.97 min and back to 5% solvent A after 3.99 min. Detection of the peptides was done by measuring the absorbance at a wavelength of 214 nm for 6 min. Calibration curves, ranging from 500 μg/ml – 1.95 μg/ml, of the non-labeled SLPs 2, 7 and 14, extracted SLPs and extraction controls, 10 mg/ml of empty liposomes spiked with 1 mg/ml SLP, were injected (10 μl/sample) into the UPLC system. The SLPs were quantified by integration of the area under the curve of the standards and extracts of the three different SLPs by using MassLynx (Waters, software 4.1.).

### ***In Vitro*** Activation of SIINFEKL Specific CD8^+^ T-Cells

*In vitro* the immunogenicity of free SLPs and liposomal SLPs was evaluated by assessing their ability to activate immature DCs that subsequently present the SIINFEKL epitope to CD8^+^ SIINFEKL specific T cells, resulting in their activation. In a 96-well flat-bottomed plate immature D1 (5 × 10^5^/well) were seeded in supplemented IMDM and incubated with either liposomal encapsulated SLP or free SLP in an equimolar, 4-step concentration range ([C] SLP: 2 μM – 0.250 μM) for 2,5 h at 37°C and 5% CO_2_. After incubation the cells were washed with supplemented IMDM to remove excess antigen (either free or encapsulated into liposomes) and T cell hybridoma B3Z cells (5 × 10^5^/well) were added and incubated overnight. The B3Z is a hybridoma CD8^+^ T cell line that is specific for the H-2 K^b^-restricted SIINFEKL epitope and contains the LacZ reporter under regulation of NF-AT element of the IL-2 promoter (11). Subsequently, ligation of the T cell receptor with the presented SIINFEKL epitope on the DC surface results in the production of the β-galactosidase protein, which was quantified in a colorimetric assay. After an overnight incubation for 12 h, the cells were incubated with chlorophenol red-β-galactopyranoside (CPRG) in a lysis buffer (PBS + 1% 18 mg/ml CPRG +0.9% 1 M MgCl_2_ + 0.125% NP40 + 0.71% 14.3 M β-mercaptoethanol) at 37°C and 5% CO_2._ The SIINFEKL minimal epitope (100 ng/ml in PBS), able to bind directly to the MHC-I complex of DCs, served as a positive control. DC that were incubated with empty liposomes and unstimulated DC were used as negative controls. Cells were incubated until the color conversion was sufficient to determine the optical density (OD) in an iMark™ mircoplate reader (Biorad, Hercules, USA) at a wavelength of 590 nm.

### Peptide Characteristics Modeling

The ranges of pIs and GRAVY indices of the SLP library were compared to those of a wide set of 24-mer peptides derived from the human genome. To determine the pI and GRAVY index ranges, 10 varying protein sequences were selected from the UniprotKB database. Several commonly expressed human household genes, tumor associated proteins and the proteins of known neoepitopes were arbitrarily selected. All theoretically possible 24-amino acid residue sequences for the different proteins were determined by making use of a Microsoft excel script. GRAVY index and pI for all predicted peptide sequences were calculated by making use of the sequence analysis tools from bioinformatics.org (12). Frequency distributions for all GRAVY indices and pIs and their corresponding graphs were made by using GraphPad Prism 7.

## Results

### Peptide Characteristics & Formulation

**Fig. 1:**
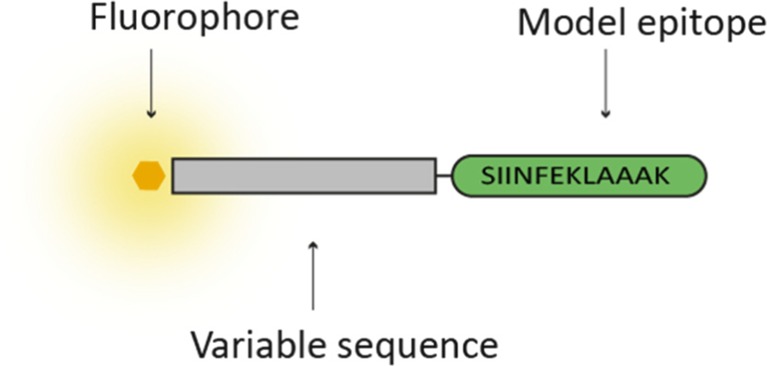
Schematic representation of the fluorescent peptides used in this study.

A total of 18 different 24-mer SLPs, widely varying in hydrophobicity and pI, were designed so that a wide range of pI and GRAVY indices were covered. The SLPs 1–15 consisted of a model epitope sequence, the ovalbumin derived SIINFEKLAAAK epitope, a variable amino acid sequence and the NBD fluorophore, which has a relative small molecular size (Mw = 165 Da) (13). The NBD fluorophore allowed for easy quantification of the SLP in the final formulation. The NBD group was conjugated to the peptide via an extra glycine, attached to a variable amino acid sequence at the N-terminal side of the epitope (Fig. 1)

. The oligo-alanine sequence at the C-terminus is well known to allow adequate C-terminal proteasome cleavage of cytotoxic T cell epitopes. Since N-terminal processing of CTL epitopes is more flexible, it is likely that the variable sequence will allow epitope processing and MHC-I presentation (14). For the SLPs 2, 7 and 14 the non-fluorescently labeled analogues were synthesized as well. For all the SLP sequences the pI and hydropathicity (GRAVY index) were calculated by making use of the sequence analysis tool from www.bioinformatics.org (Table I).

In order to formulate the SLPs into cationic liposomes, the SLPs need to be fully dissolved. Because of the wide variety of pI and GRAVY values, this turned out to be not possible with a single solvent. Based on several exploring experiments, the following solvents were used: ACN:H_2_O (1:1 v/v; solvent A), CHCl_3_:MeOH:H_2_O (60:36:4, v/v; solvent B) and 0.04% (*w*/*v*) NH_4_OH (solvent C). The SLPs 1–6 were dissolved in solvent A, SLPs 7–10 in solvent B and SLPs 11–15 in solvent C. SLPs 11 and 13 did not dissolve in 0.04% NH_4_OH, however, preparing a concentrated stock solution in DMSO of the SLPs followed by a 20-fold dilution in 0.04% NH_4_OH resulted in peptide dissolution. Depending on the solvent type, the SLP solution was added either to the lipid mixture prior to roto-evaporation (solvent B) or to the dry lipid film during the rehydration step (solvent A and C). In the case of the aqueous solvents, the pH is preferably higher than the pI, to promote electrostatic interaction between the negatively charged SLPs and the quaternary amine group of DOTAP, which has a permanent positive charge over the whole pH range (7,16). Therefore, the pH of solvent A was adjusted to a value of 8.5 after peptide dissolution. In the case of solvent C, no further pH adjustments were required, since the solvent by itself is already basic (pH = 9.5). The SLPs that were mixed with the lipids prior to roto-evaporation formed a homogeneous dry lipid-peptide film. During the formulation process, the SLPs were formulated at 1 mg/ml, except for SLP 1, which was formulated at 0.5 mg/ml because of solubility problems at higher concentrations.

### Physicochemical Characteristics of the Liposomes

**Fig. 2:**
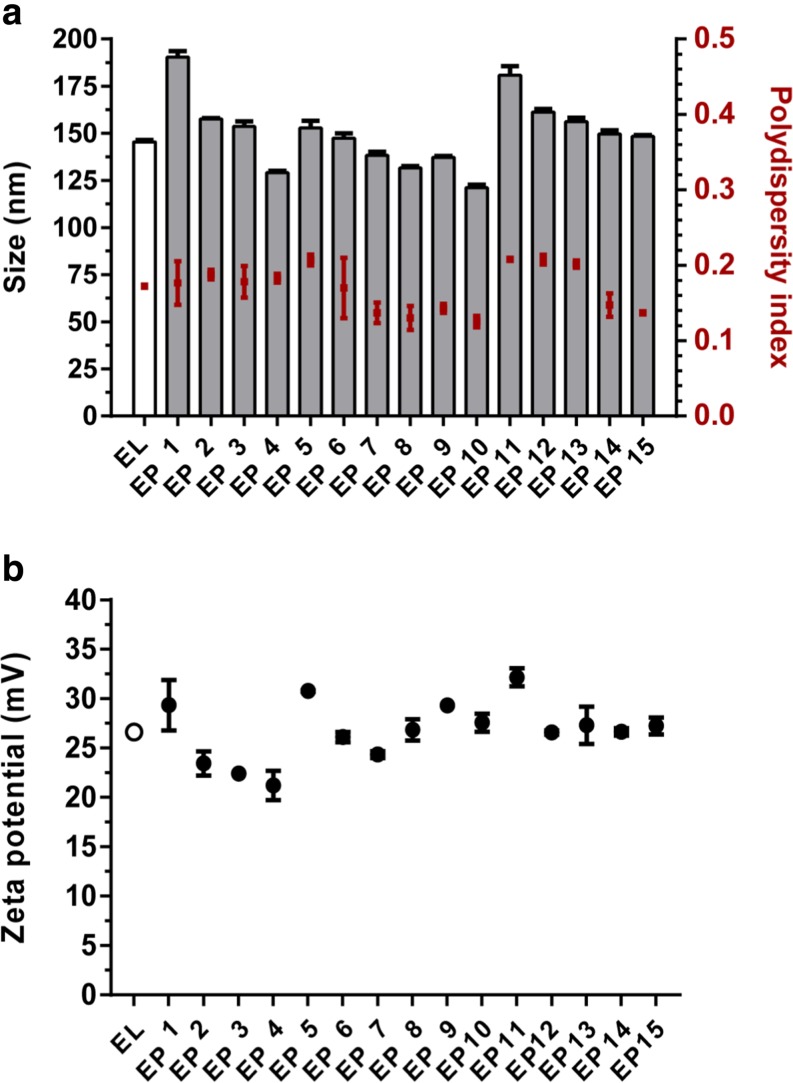
Physicochemical characteristics of empty liposomes (EL) and liposomal encapsulated peptides (EP). The fifteen SLP loaded liposomal formulations had a comparable hydrodynamic diameter, polydispersity index (**a**) and zeta potential (**b**). Data is represented as mean ± SD (*n* = 3).

Empty liposomes and the 15 SLP loaded liposomes had comparable hydrodynamic diameters (Z-average < 200 nm), were fairly monodisperse (PDI <0.22) and positively charged (zeta potential ±26 mV) (Fig. 2). Only small differences in liposome characteristics were observed between the different liposomal SLP formulations, i.e., particle characteristics appeared to be fairly independent of the loaded SLP or the used solvent. Some of the SLP loaded liposomes had a slightly lower positive zeta potential (Fig. 2), possibly due to the characteristics of the individual SLP. Storage of the liposomes for 8 weeks at 4°C did not detectably affect Z-average, PDI or zeta potential, indicating that all SLPs were formulated into colloidally stable liposomal dispersions (data not shown).

The fifteen different SLPs were all successfully loaded into the DOTAP:DOPC liposomes (Table II). The majority of the formulations (*n* = 13) were comparable regarding their encapsulation efficiency, indicating that the liposomes are able to accommodate a wide range of different SLPs. In peptide recovery, however, more variation was observed, reflecting variable losses during the production process steps, which apparently depends on the physicochemical properties of the SLP.

**Table II:** Loading Efficiency and Peptide Recovery of all Liposomal Formulated NBD Labeled SLPs

Peptide ID	Peptide solvent	Encapsulation efficiency (%)	Peptide recovery (%)
1		61	9
2		41	10
3	ACN:MQ	62	7
4		61	6
5		40	7
6		46	15
7		51	35
8	CHCl_3_:MeOH:MQ	56	10
9		35	8
10		32	5
11		79	21
12		20	6
13	0.04% NH_4_OH	65	7
14		9	4
15		17	5

To determine the potential influence of the fluorophore on the liposomal characteristics, three non-fluorescently labeled SLPs were formulated as well.

The non-labeled analogues of peptides 2, 7 and 12 were chosen in such a way that all three different production solvents were used. Encapsulation of the three SLPs yielded liposomes that had comparable physicochemical characteristics (Z-average, PDI and zeta potential) to those containing the fluorescently labeled counterparts (Z-average < 200 nm, PDI <0.25 and zeta potential ± 26 mV). However, the non-labeled SLPs showed for all three different formulation strategies an adequate encapsulation efficiency while a generally higher peptide recovery was observed (Table III).

**Table III:** Loading Efficiency and Peptide Recovery for Three Non-Labeled Analogues of SLPs 2, 7 and 14. The Encapsulation Efficiency and Peptide Recovery of the Three Fluorescently Labeled SLP Analogues are Depicted in Italics Between Brackets

Peptide ID	Peptide solvent	Encapsulation efficiency (%)	Peptide recovery (%)
2	ACN:MQ	36 *(41%)*	24 *(9%)*
7	CHCl_3_:MeOH:MQ	37 *(51%)*	60 *(35%)*
14	0.04% NH_4_OH	29 *(9%)*	16 *(4%)*

### ***In Vitro*** CD8^+^ T-Cell Activation

**Fig. 3:**
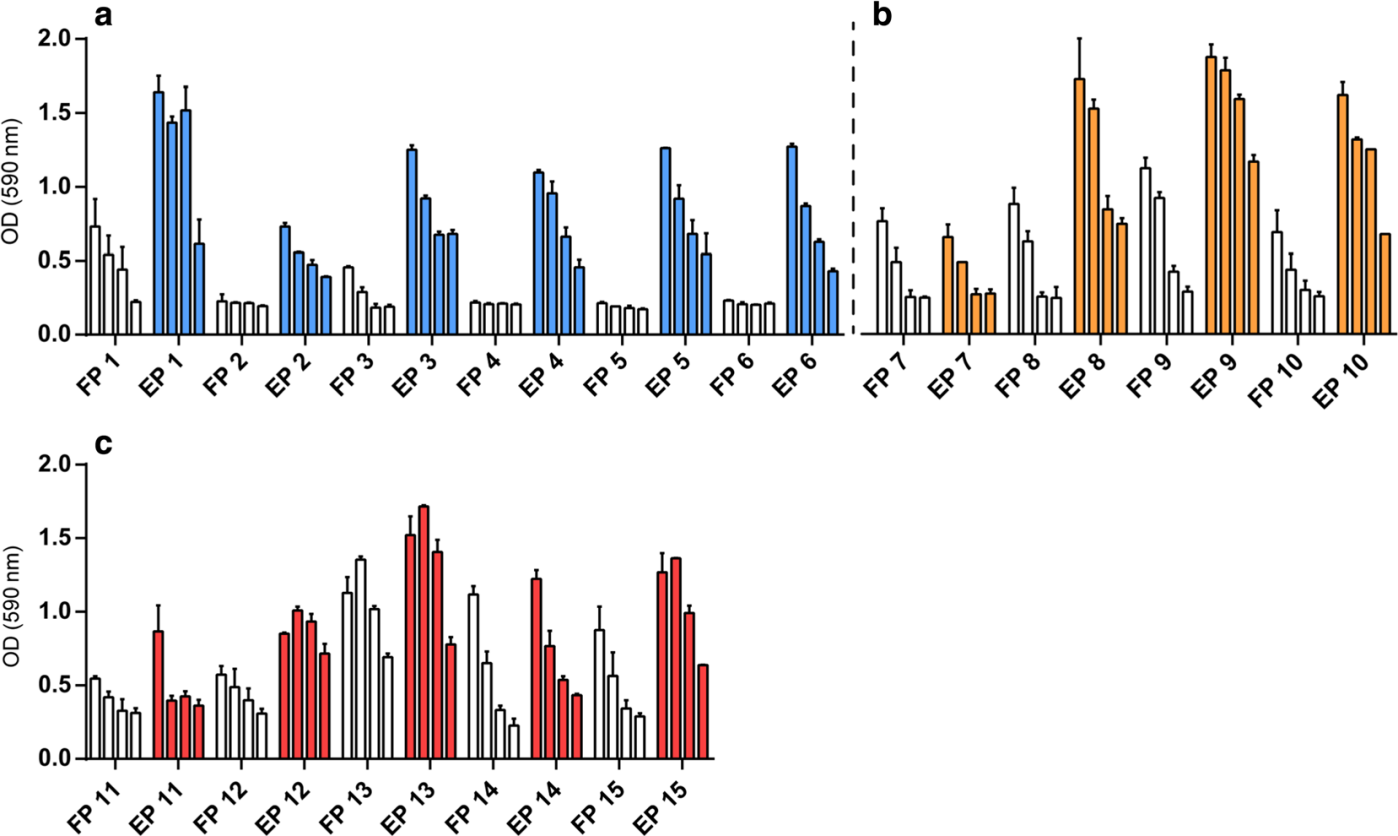
*In vitro* activation of the SIINFEKL-specific hybridoma CD8^+^ T cells (B3Z). DCs were incubated during 2.5 h with titrated amounts [2–0.250 μM] of either free SLP (FP) or liposomal encapsulated SLP (EP) produced with solvent A. (**a**), solvent B (**b**) or solvent C (**c**). The DCs were washed and co-cultured overnight with the CD8^+^ T cells. Graphs depict T cell activation based on optical density determined (OD) at 590 nm. Data is represented as mean with range.

The immunological properties of the formulated peptides were assessed by monitored antigen presentation by murine DCs using the SIINFEKL specific CD8^+^ T cell line B3Z *in vitro*. DCs are able to activate the CD8^+^ T cells after SLP uptake, processing and cross-presentation of the SIINFEKL epitope in their MHC class I molecules (11). In fig. 3 is shown that 10 out of 15 formulations outperformed the respective free SLPs, while 2 out of 15 SLPs showed similar levels of T cell activation. For the remaining 5 formulations similar levels of CD8^+^ T cell activation were observed for the liposomal SLP and the free SLP. These data indicate that the used encapsulation methods did not compromise the immunological properties of SLPs in the final formulations. Between the three subsets of peptides an increasing B3Z activation was observed for SLPs with a more lipophilic character (reflected by a high GRAVY index). Cell viability during the assay conditions was controlled by presentation of the SIINFEKL minimal epitope and empty liposomes served as a negative control (Supplementary Fig. 1).

### Theoretical Distribution of Physicochemical Properties of Naturally Occurring 24-Mer Peptides Derived from the Human Genome

**Fig. 4:**
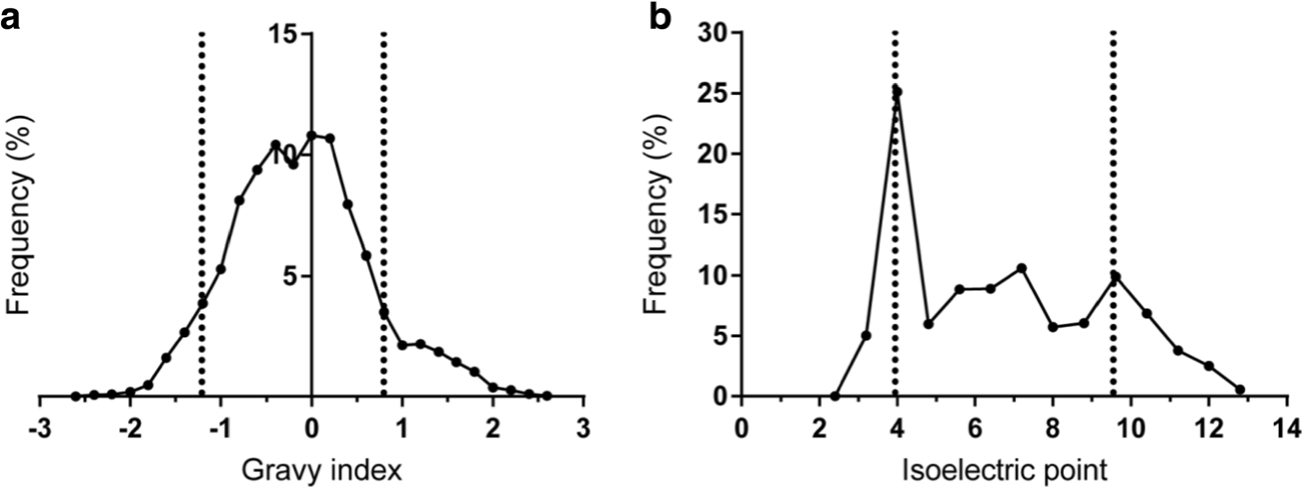
Frequency distribution of 5546 possible 24-mer peptide sequences that are present in a total of 10 different arbitrarily selected human proteins. Dotted lines represent the lower and upper limit of the (**a**) GRAVY index and (**b**) isoelectric point range of the encapsulated SLPs. For the GRAVY index 85.5% and for the pI 69.6% of the predicted peptides are in the range of the tested SLP candidates used for liposomal encapsulation.

In order to determine the applicability of cationic liposomes for encapsulation of human protein-derived SLPs in general, the GRAVY index and pI values of the encapsulated SLP library were compared to those of a large set of naturally occurring 24-mer peptide sequences. For a total of 10 different representative proteins, all theoretical possible 24-mer long peptides (*n* = 5546) were determined (alternative splicing variants were not taken into account). Peptide sequences were determined by dissecting the protein sequence into blocks of 24 amino acids in a sequential order (i.e., 1–24, 2–25, etc.). The included proteins were arbitrarily selected and include human melanoma antigens, neo-epitopes, and several commonly expressed human proteins to generate a wide range of different peptides (Supplementary Table 1). The protein sequences were derived from the Uniprot database and all the 24-mer peptide sequences of each protein were listed by making use of an excel script, after which their pI and GRAVY index was determined. The majority of the resulting peptide sequences (85.5%, *n* = 4742) fall within the GRAVY range of our peptide library ([−1.208] – [0.771]) whilst for the pI range this was 69.6% (*n* = 3860) (3.95–9.54) (Fig. 4).

## Discussion

In this study we investigated the potential of DOTAP-based liposomal formulations for the loading of SLP antigens. Cationic DOTAP:DOPC liposomes are known for their ability to enhance DC maturation, compared to negatively charged or neutral liposomes, and are able to improve the subsequent T cell priming when combined with an epitope-based SLP (17,18). The production of stable liposomes is not possible with only cationic lipids and therefore a neutral lipid, DOPC, is included to stabilize the liposomes (19). The potential of the DOTAP:DOPC liposomes as a vaccine delivery platform for therapeutic cancer vaccines has recently been shown (4,7). However, the general applicability of cationic liposomes for the encapsulation of SLPs with a large variety of physicochemical properties has not been reported yet. This flexibility is an essential requirement for a generic delivery system to accommodate personalized neoepitope-based peptide vaccines, since they will consist of a range of variable SLPs (1,2,5). By making use of a library of fifteen SLPs, widely varying in pI and GRAVY index values, the feasibility of our liposomal delivery system for SLP-based vaccines was analyzed in detail.

In order to load SLPs into the cationic liposomes, we made use of the previously optimized dehydration-rehydration protocol for SLP encapsulation (4,7). In the current study we applied variations to this protocol in order to enable the encapsulation of a wide range of physicochemically distinct SLPs. A main challenge during the formulation of some of these SLPs was to completely dissolve them prior to their encapsulation. Previously reported data on the DOTAP:DOPC liposomes loaded with the so-called “OVA24” SLP, harboring the SIINFEKL epitope, showed that a decrease of the rehydration solvents’ pH below the pI of the SLP led to a significant reduction of the loading efficiency (7), suggesting that the entrapment of OVA24 in the cationic liposomes is highly dependent on electrostatic interactions. Because of the cationic nature of the liposomes, the SLP encapsulation might be a combination of entrapment and SLP adsorption on the liposomal surface. However, since the zeta-potentials of SLP loaded liposomes were comparable to those of empty liposomes, we assume that the majority of the SLPs was located inside the liposomes. Although the different preparation methods for the loading of the hydrophilic and hydrophobic SLPs might influence the physicochemical characteristics of the particles, we did not observe major differences between the loaded liposomes with regard to their size, charge and polydispersity. However, for the NBD conjugated SLPs some variation was seen regarding loading efficiency and peptide recovery. The three non-labeled control peptides appear to perform even better in these aspects. In the current study small batch volumes were produced, which could have contributed to a relatively high loss of SLPs during manufacture. This may be overcome by the use of larger batch volumes and smaller production equipment. This likely will reduce the relative loss of SLP due to reduction of dead volumes and adsorption of SLP and/or lipids to surfaces during the formulation process, such as glassware and filters. The higher peptide recoveries for the non-labeled SLPs are most likely due to the absence of the NBD group, possibly reducing the amount of lost SLP due to adsorption.

In an *in vitro* setting DCs were able to activate SIINFEKL specific CD8^+^ T cells after they were incubated with the SLP loaded liposomes. These results indicate that the encapsulation process did not hamper the immunological properties of the SLPs and that the liposomes did not interfere with the process of antigen processing and cross-presentation by the DCs. Moreover, as compared to free SLPs, liposomal delivery of SLPs seems to promote antigen cross-presentation. The SLPs with a GRAVY index >0 showed higher levels of CD8^+^ T cell activation compared to the SLPs with lower GRAVY values (Table I, supplementary Fig. 2). This could be caused by differences in antigen processing due to the different amino acid sequences or an altered mechanism of SLP uptake by the DCs. The more hydrophobic SLPs could potentially form additional supramolecular structures, e.g., micelles, influencing their uptake and subsequent processing by DCs (20,21). Therefore, when assessing the effect of SLP encapsulation in cationic liposomes, the influence of the variable sequence on the levels of CD8^+^ T cell presentation was excluded by comparing the free peptides with their liposomal equivalent for each individual peptide. Furthermore, although we only studied the immunogenicity *in vitro*, in our previous work we have shown the potential of liposomal SLPs for cancer immunotherapy *in vivo* (4,7).

The data in this study show the applicability of DOTAP-based liposomes as a flexible delivery system with intact immunological activity for fifteen SLPs which widely differ in physicochemical characteristics. The comparison of characteristics, GRAVY index and pI, of the used SLP library to a wide range of human based peptides showed a large overlay, indicating that the majority of these peptides are potential candidates for liposomal encapsulation.

## Conclusion

In this study the separate encapsulation of an representative array of physicochemical distinct different SLPs in DOTAP:DOPC liposomes was described. The physicochemical properties of the SLP-loaded liposomes were characterized and their ability to activate CD8^+^ T-cells after engulfment and antigen presentation by dendritic cells was assessed. The results show that the proposed formulation strategy, the dehydration-rehydration method, in combination with three solvent/encapsulation variants, is a feasible strategy to encapsulate physicochemically widely different SLPs on a small scale with preserved immunogenicity. Hereby we underscore the potential of DOTAP-based liposomes as a flexible particulate delivery system for peptide based cancer vaccines**.** Based on the current study and our previous work, we are now focusing on preclinical development of DOTAP-based neoepitope peptide vaccines.

## Electronic supplementary material

ESM 1 (DOCX 239 kb)

## Abbreviations

DC: Dendritic cell
OD: Optical density
PB: Phosphate buffer
PDI: Polydispersity index
SLP: Synthetic long peptide
