# Supplementary material for: Cationic Liposomes: A Flexible Vaccine Delivery System for Physicochemically Diverse Antigenic Peptides
Source: Pharm Res. 2018 Sep 12;35(11):207. doi: 10.1007/s11095-018-2490-6 (PMC6156754; doi:10.1007/s11095-018-2490-6)
Supplement: Supplementary file 1 — (DOCX 239 kb) [file 11095_2018_2490_MOESM1_ESM.docx]

**Supplementary data**

**
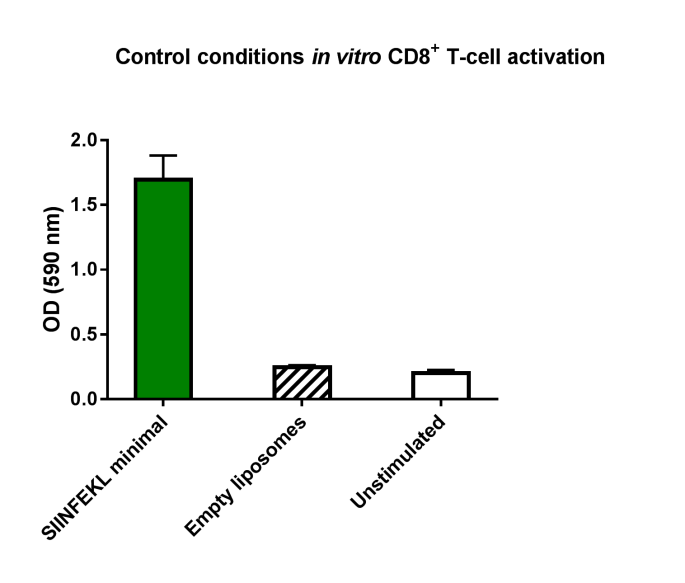
**

Supplementary figure 1 The SIINFEKL minimal epitope (100 ng/ml in PBS) served as a positive control. DCs that were incubated with empty liposomes and unstimulated DCs were used as negative controls. Data is represented as mean $\boldsymbol{\pm}$ SD (n=4).

Supplementary table 1 Proteins used for the peptide prediction

| **Protein** | **Number of derived 24-mer peptides** | **pI range** | **GRAVY range** |
| --- | --- | --- | --- |
| GAPDH | 385 | 3.38 – 12.50 | [-1.788] – [0.837] |
| GCPR | 882 | 3.26 – 12.50 | [-1.804] – [2.267] |
| LDLR | 834 | 3.05 – 12.22 | [-1.688] – [2.592] |
| MART-1 | 98 | 3.61 – 11.94 | [-1.746] – [1.775] |
| TRP-1 | 769 | 3.17 – 12.52 | [-2.383] – [2.237] |
| GP100 | 637 | 3.17 – 12.52 | [-1.596] – [2.479] |
| IgG heavy chain | 306 | 3.64 – 10.57 | [-1.746] – [-0.992] |
| ADPGK | 476 | 3.23 – 12.80 | [-1.071] – [1.662] |
| DPAGT | 385 | 3.33 – 12.50 | [-1.175] – [2.404] |
| REPS-1 | 774 | 3.03 – 12.82 | [-2.125] – [0.883] |


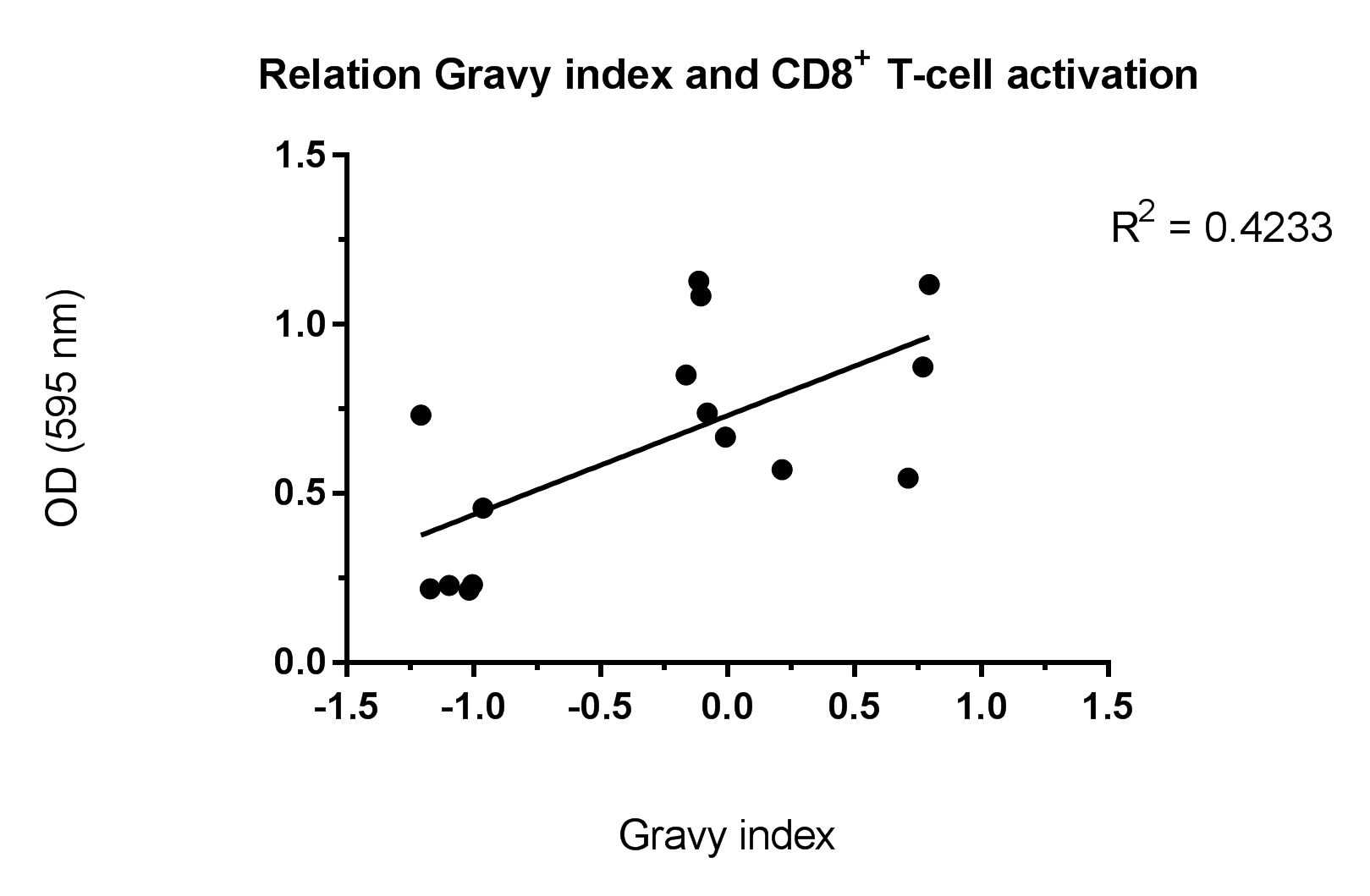


Supplementary figure 2 Correlation plot of the observed CD8^+^ T-cell activation by free SLPs and their Gravy index. Each dot represents the B3Z activation (expressed as OD at 595 nm) resulting from a free SLP dose of 2 μM.
